# Evaluation of the paraoxonase-1 kinetic parameters of the lactonase activity by nonlinear fit of progress curves

**DOI:** 10.1080/14756366.2019.1695792

**Published:** 2019-12-02

**Authors:** Marko Goličnik, Aljoša Bavec

**Affiliations:** Institute of Biochemistry, Faculty of Medicine, University of Ljubljana, Ljubljana, Slovenia

**Keywords:** Paraoxonase-1, progress–curve analysis, nonlinear regression, Lambert W function

## Abstract

Although paraoxonase-1 (PON1) activity has been demonstrated to be a reliable biomarker of various diseases, clinical studies have been based only on relative comparison of specific enzyme activities, which capture differences mainly due to (usually unknown) PON1 concentration. Hence, the aim of this report is to present for the first time the simple evaluation method for determining autonomous kinetic parameter of PON1 that could be also associated with polymorphic forms and diseases; i.e. the Michaelis constant which is enzyme concentration independent quantity. This alternative approach significantly reduces the number of experiments needed, and it yields the results with great accuracy.

## Introduction

Paraoxonase-1 (PON1) is a key Ca^2+^-dependent enzyme of liver-derived high-density lipoproteins (HDLs), which is linked to various pathophysiological disorders^1^. Although the “physiological” substrates of PON1 appear to be lactones, PON1 has a large number of promiscuous activities towards organophosphate triesters, arylesters, thiolactones and cyclic carbamates^2^. Thus, PON1-status can be determined by measuring enzyme activity levels with different substrates, and the latter can vary by over 10-fold between individuals with various PON1 genetic polymorphisms and pathological conditions^3^^,^^4^.

The most convenient method for determining PON1-status and functional phenotype is based on 2-dimensional enzyme activity plot that displays rates of diazoxonase versus paraoxonase activity^5^^,^^6^. As this 2-substrate assay/plot technique involves the use of two highly toxic organophosphates, identification and characterisation of non-toxic discriminatory substrates for determination of PON1 activity still excites an interest among clinical researchers^7^. Although current assays may provide substantial diagnostic “PON1 status” values, they are constrained only to the specific enzyme activity measurements which are unavoidably affected by (unknown) PON1 concentration and its affinity to a substrate. Even though if one PON1 alloform has a lower limiting rate *V*_max_, but it has higher affinity (i.e. lower Michaelis constant *K*_m_) for a particular substrate, then the catalytic efficiency (*V*_max_/*K*_m_) and consequently specific enzyme activity measurements of both PON1 alloforms can show nearly the same values. Further, another enzyme can form ternary complex with HDL and PON1; i.e. myeloperoxidase (MPO) which produces highly reactive oxidant hypochlorous acid (HOCl). MPO oxidises PON1 on tyrosine 71, a residue found that is critical for HDL binding which impairs catalytic abilities of PON1^8^; i.e. substrate affinity to the enzyme active site and its turnover number. Although the latter cannot be determined when active enzyme concentration is unknown, the concentration independent *K*_m_ value can be more informative and consistent with true PON1 status. Thus, only an evaluation of complete set of PON1 kinetic parameters can provide predictive and conclusive evidence-based use of it as biomarker in health and disease.

## Materials and methods

### Study design

As our study was designed only for the development of simple and quick methodology for determination of PON1 kinetic characteristics, we selected leftover routine blood samples of six healthy blood-donors. Since the biological material used in this report were obtained from leftover routine specimens, and the samples were not used for any other particular study, informed consent from volunteers and ethical approval was unnecessary because the samples were no longer traceable.

### Methods

**Figure 1.**
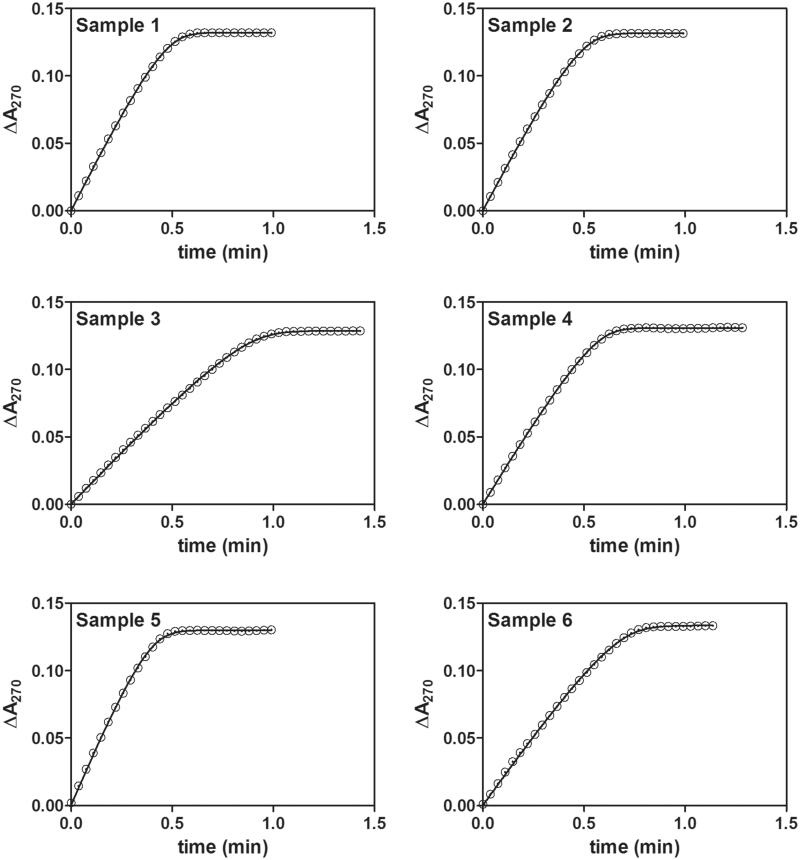
Fit of kinetic progress curve data for each sample. Symbols represent absorbance readings at the given reaction time. Only one progress curve per sample and only half of the data points per progress curve are shown, for clarity. Smooth lines represent least-square model curves generated by fit of the theoretical equation Equation (2) with the parameter values obtained by modified model (GraphPad, Lambert W) shown in Table 1.

Blood samples were collected in heparin containing tubes, and the cells were removed by centrifugation for 10 min at 2000**×***g*. Plasma samples were afterward stored in microtubes at 4 °C. Lactonase hydrolytic activities of PON1 with dihydrocoumarin (Δε_270_ = 1295 M^−1^ cm^−1^) were measured spectrophotometrically at 270 nm^2^ within 4 h after the blood sample collection. In an assay the cuvette contained 100 μM substrate in 50 mM Tris/HCl (pH 8, 1 mM CaCl_2_, and 1% of methanol) in a total volume of 2 ml. The reaction was initiated by the addition of 20 μL of plasma, and full progress curve runs were carried out in less than 2 min; i.e. unless the entire substrate was converted to product (Figure 1). The measurements for each sample were carried in duplicate or triplicate.

### Data analysis

Progress curve experimental data were analysed by nonlinear regression fitting programme Dynafit using either numerical integration of differential equations that describe simple Michaelis–Menten reaction model E + S ⇆ ES → E + P or newly described algebraic solution for the Michaelis–Menten equation, which includes the Lambert *W*(*x*) function^9^^,^^10^:

(1) $${\left[P\right]}_{t}={\left[S\right]}_{0}-K_{m}\cdot W_{0}\left\{\frac{{\left[S\right]}_{0}}{K_{m}}\cdot \;\text{exp}\;\left(\frac{{\left[S\right]}_{0}-V_{\max}\cdot t}{K_{m}}\right)\right\}$$

*V*_max_ and *K*_m_ in Equation (1) represent the limiting velocity and Michaelis constant, respectively. Finally, the approximation to the Lambert W function in Equation (1) for the product accumulation was also implemented into the GraphPad Prism (GraphPad Software, San Diego, California, USA) software package as a user-defined built-in explicit model equation for calculating model curves^11^:

(2) $${\left[P\right]}_{t}={\left[S\right]}_{0}-K_{m}\cdot \left(1.45869\cdot \;\text{ln}\;\left(1.2\cdot \frac{x}{\;\text{ln}\;\left(2.4\cdot \frac{x}{\;\text{ln}\;\left(1+2.4\cdot x\right)}\right)}\right)-0.45869\cdot \;\text{ln}\;\left(2\cdot \frac{x}{\;\text{ln}\;\left(1+2\cdot x\right)}\right)\right)$$

where

(3) $$x=\frac{{\left[S\right]}_{0}}{K_{m}}\cdot \;\text{exp}\;\left(\frac{{\left[S\right]}_{0}-V_{\max}\cdot t}{K_{m}}\right)$$

## Results and discussion

The individual time–course data for each sample (Figure 1), and the nonlinear regression analyses in terms of different computing approaches give the fitted theoretical curves for the Michaelis–Menten reaction model. Progress curves were first analysed by numerical integration approach, which has been the most widely used method for time–course data analysis among enzymologists (Table 1, DynaFit numerical integration). Therefore, the best fit to the time–product concentration data obtained by performing DynaFit numerical integration programme yielded the reference values *K*_m_, *V_max_* and [*S*]_0_ (see Table 1). However, the latter numerical approach can have drawbacks that are widely ignored and show several weak points^12^, although the most common computing method to progress–curve analysis these days is to use numerical integration solvers linked to nonlinear regression algorithms. Hence, we also estimated the kinetic parameters in terms of an algebraic approach that gives the direct solution of Equation (1) to the integrated Michaelis–Menten rate equation (Table 1, DynaFit Lambert W)^9^. Unfortunately, the requirement for the Lambert *W*(*x*) built-in function in software (e.g. Maplesoft Maple, Mathworks Matlab, BioKin DynaFit) in this alternative explicit expression to the integrated model rate equation for computing product accumulation with Equation (1) is another drawback as the most available curve-fitting software packages (e.g. Microsoft Excel, GraphPad Prism, Systat SigmaPlot) are not set up to handle equations that involve W(x), and hence they are of none use for direct time–course data analysis in terms of Equation (1). To avoid this problem, we finally used the solution for [*P*]*_t_* based on an exact approximation Equation (2), which is elementary and consequently easily implemented in any standard software^11^ and calculated kinetic parameters from progress curves (Table 1, GraphPad Lambert W). Figure 1 shows a fit of the reaction progress curves data in GraphPad Prism by using Equation (2); i.e. the approximation to the Lambert W function in Equation (1). Table 1 summarises all values of the fitted estimates of the kinetics parameters where the best parameter values yielded almost identical good fits to the experimental data for all of the computing methods. The given values are also in agreement with the results obtained by the traditional double-reciprocal Lineweaver-Burk plots methodology^2^.

**Table 1.** Parameters aquired by progress–curve fitting. Comparison of fitted values obtained using the numerical integration approach (*DynaFit*, see Ref. [9]), the exact algebraic model Equation (1) with the Lambert W(x) function (*DynaFit*, see Ref. [9]), and the approximation of W(x) of the modified model Equation (2) (*GraphPad, Lambert W*, see Ref. [11]). The measurements for each sample were carried in duplicate or triplicate. Data are means ± SD.

	DynaFit numerical integration	DynaFit Lambert W	GraphPad Lambert W		DynaFit numerical integration	DynaFit Lambert W	GraphPad Lambert W
	Sample 1				Sample 2		
[S]_0_ (μM)	*101.0 ± 0.1*	101.0 ± 0.1	100.9 ± 0.1	[S]_0_ (μM)	*100.6 ± 0.1*	100.6 ± 0.1	100.6 ± 0.1
K_m_ (μM)	*11.3 ± 0.5*	11.3 ± 0.5	11.0 ± 0.5	K_m_ (μM)	*11.8 ± 0.6*	11.8 ± 0.6	11.5 ± 0.5
*V*_max_ (μM/min)	*250.2 ± 2.1*	222.3 ± 8.6	248.8 ± 1.8	*V*_max_ (μM/min)	*241.3 ± 2.3*	204.5 ± 8.6	239.9 ± 2.1
	Sample 3				Sample 4		
[S]_0_ (μM)	*98.3 ± 0.2*	98.3 ± 0.2	98.3 ± 0.1	[S]_0_ (μM)	*99.9 ± 0.1*	99.9 ± 0.1	99.9 ± 0.1
K_m_ (μM)	*8.6 ± 0.5*	8.6 ± 0.5	8.4 ± 0.4	K_m_ (μM)	*9.5 ± 0.3*	9.5 ± 0.3	9.2 ± 0.1
*V*_max_ (μM/min)	*129.2 ± 1.1*	150.5 ± 7.4	128.6 ± 1.0	*V*_max_ (μM/min)	*204.3 ± 1.1*	214.7 ± 6.1	202.9 ± 0.3
	Sample 5				Sample 6		
[S]_0_ (μM)	*99.2 ± 0.2*	97.6 ± 0.1	99.2 ± 0.2	[S]_0_ (μM)	*101.9 ± 0.3*	101.1 ± 0.2	101.9 ± 0.2
K_m_ (μM)	*13.8 ± 1.2*	11.4 ± 0.6	13.5 ± 1.1	K_m_ (μM)	*10.7 ± 0.9*	9.6 ± 0.6	10.4 ± 0.8
*V*_max_ (μM/min)	*304.2 ± 5.7*	252 ± 10	302.6 ± 5.2	*V*_max_ (μM/min)	*176.5 ± 2.4*	179.5 ± 9.7	175.5 ± 2.1

PON1 enzyme levels can range widely even between individuals with the same PON1 genotypes, and hence PON1 status which considers both PON1 genotypes and PON1 activity is a more informative for use in epidemiological studies then PON1 genotype alone^1^^,^^3^. Due to the relatively high concentrations of PON1 in human blood, many studies have also been performed on the inhibitory effect of medical drugs^13–16^ or metal elements^17^ on PON1 activity. Recent studies have shown that there is also a specific interaction of MPO-apoAI-PON1 on HDL surface that seems to be germane to the development of different diseases^8^. Therefore, PON1 studies widely utilise various assays for determination of PON1 phenotypes whenever possible, but unfortunately they are usually limited only on the specific enzyme activity measurements, and mostly with highly toxic substrates^3–6^.

Although basic biochemical and physiological principles dictate that it is the activity of a given enzyme that is important with respect to its function, two kinetic parameters determine the activity of enzyme at given substrate concentration; i.e. limiting rate *V*_max_ (=*k*_cat_*·[E]_T_*) which depends on turnover number and enzyme active sites concentration, and the Michaelis constant *K*_m_ which is not concentration dependent characteristics of an enzyme. However, the values of *K*_m_ are associated with polymorfic forms^2^, enyzme modifications and its environment.

Traditionally, the quantitative kinetics of enzyme-catalyzed reactions have been studied in terms of the correlation between initial rates and substrate concentrations according to the hyperbolic Michaelis–Menten equation. Although direct analysis that use Michaelis–Menten equation is easy to perform by various nonlinear regression curve-fitting programmes, the initial-velocity measurements still require a high number of individual experiments, due to the high sensitivity of the reaction rates to noise^18^. On the other hand, analyses of complete progress curves can provide the same information, although this can be achieved with a single experimental assay that measures the kinetics data at every concentration between the initial value and that at the end of the reaction.

**Figure 2.**
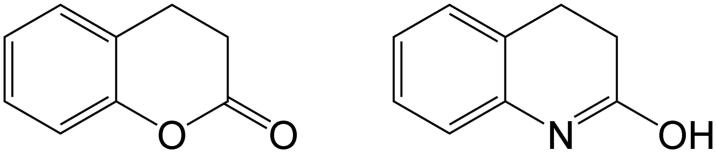
Paraoxonase-1 lactone substrate dihydrocoumarine (left) and its competitive inhibitor 2-hydroxyquinoline (right).

However, the choice of the substrate is crucial for practical single progress–curve assays. The required experimental condition for the independent estimation of both *K*_m_ and *V*_max_ from a progress curve is the initial substrate concentration which must be of the same order of magnitude as *K*_m_^12^_,_ and the substrates with high *K*_m_ values are not usable for such assays. Consequently, we decided for measuring of the PON1 lactonase activity with dihydrocoumarine which is non-toxic and shows low but significant twofold difference of *K*_m_ values for the most widely PON1 isoforms (*K*_m_ = 22 μM for Q-type, *K*_m_ = 13 μM for R-type, see Ref. [2]) Consequently, we were also able to avoid the problems with insolubility and spontaneous hydrolysis with the use of low initial substrate concentration. It should be remembered that structuraly mimetic molecule 2-hydroxyquinoline (Figure 2) specifically inhibits enzymatic activities of PON1 with low *K*_i_ (∼2 μM)^2^. However, since there evidently are two PON1 isoforms present, the assay yields an averaged *K*_m_ value in a case of heterozygote, meaning for correct PON1 status both, genotype and PON1 activity should be followed.

In conclusion, traditional *K*_m_ determining double-reciprocal Lineweaver-Burk approach is not applicable when dealing with hundreds of biological samples in clinical diagnostic or high throughput screening drug research. This study provided an opportunity to carefully examine challenges that may exist when using quick and safe PON1 single progress–curve measurements with non-toxic substrate, and simple direct calculation of kinetic parameters from raw time–concentration data by performing any available nonlinear curve-fitting approach and software like MS Excel^19^. We believe that prompt and accurate evaluation of both kinetic parameters *K*_m_ and *V*_max_ is essential for determination of any enzyme status and provides more information than genotype and specific activity of the enzyme alone.
